# A Single Nucleotide Deletion in an ABC Transporter Gene Leads to a Dwarf Phenotype in Watermelon

**DOI:** 10.3389/fpls.2019.01399

**Published:** 2019-11-13

**Authors:** Huayu Zhu, Minjuan Zhang, Shouru Sun, Sen Yang, Jingxue Li, Hui Li, Huihui Yang, Kaige Zhang, Jianbin Hu, Dongming Liu, Luming Yang

**Affiliations:** College of Horticulture, Henan Agricultural University, Zhengzhou, China

**Keywords:** watermelon, dwarf, BSA-seq, SSR marker, fine mapping, ABC transporter

## Abstract

Dwarf habit is one of the most important traits in crop plant architecture, as it can increase plant density and improved land utilization, especially for protected cultivation, as well as increasing lodging resistance and economic yield. At least four dwarf genes have been identified in watermelon, but none of them has been cloned. In the current study, the *Cldw-1* gene was primary-mapped onto watermelon chromosome 9 by next-generation sequencing-aided bulked-segregant analysis (BSA-seq) of F_2_ plants derived from a cross between a normal-height line, WT4, and a dwarf line, WM102, in watermelon. The candidate region identified by BSA-seq was subsequently validated and confirmed by linkage analysis using 30 simple sequence repeat (SSR) markers in an F_2_ population of 124 plants. The *Cldw-1* gene was further fine-mapped by chromosome walking in a large F_2_ population of 1,053 plants and was delimited into a candidate region of 107.00 kb. Six genes were predicted to be in the candidate region, and only one gene, *Cla010337*, was identified to have two single nucleotide polymorphisms (SNPs) and a single nucleotide deletion in the exons in the dwarf line, WM102. A derived cleaved amplified polymorphic sequence (dCAPS) marker was developed from the single nucleotide deletion, co-segregated with the dwarf trait in both the F_2_ population and a germplasm collection of 165 accessions. *Cla010337* encoded an ATP-binding cassette transporter (ABC transporter) protein, and the expression levels of *Cla010337* were significantly reduced in all the tissues tested in the dwarf line, WM102. The results of this study will be useful in achieving a better understanding of the molecular mechanism of the dwarf plant trait in watermelon and for the development of marker-assisted selection (MAS) for new dwarf cultivars.

## Introduction

Cultivated watermelon (*Citrullus lanatus* var. *lanatus* (Thunb.) Matsum. & Nakai) is an important horticultural crop in the Cucurbitaceae family and is one of the most widely consumed fresh fruits worldwide. The diploid genome of watermelon is relatively small (∼425 Mb; Guo et al., 2012), consisting of twenty-two chromosomes (2n = 2x = 22). The draft genome of the East Asian watermelon cultivar 97103 was sequenced and assembled in 2012 using next-generation sequencing (NGS) technology (Guo et al., 2012). A large number of SSR and insertion/deletion (Indel) markers have been developed from the assembly of the watermelon genome (Ren et al., 2012; Zhu et al., 2016). The rapid development of genetic and genomic resources in watermelon was greatly facilitated by gene and QTL mapping. A number of genes controlling important traits in watermelon have been mapped and cloned, such as fruit shape (Dou et al., 2018), flesh color (Zhang et al., 2017), the tonoplast sugar transporter gene (*ClTST2*) (Ren et al., 2018), and lobed leaf shape (Wei et al., 2017), as well as QTLs associated with resistance to different pathogens (Lambel et al., 2014; Kim et al., 2015; Ren et al., 2015; Branham et al., 2017).

Dwarf plant habit is an important trait in plant breeding, as reduced height is associated with lodging tolerance, increased economic sink size, and stable yield increases in many crops. A number of genes controlling plant height have been identified and mapped in the Cucurbitaceae family. To date, at least five genes have been identified for compact growth habit or dwarfism-related plant architecture in cucumber, including *compact* (*cp*) (Kauffman and Lower, 1976), *cp-2* (Kubicki et al., 1986), *supercompact* (*scp*) (Niemirowicz-Szczytt et al., 1996), *scp-1* (Wang et al., 2017), and *scp-2* (Hou et al., 2017). The *cp* mutant was fine-mapped to a 220 kb region at the end of chromosome 4 in cucumber (Li et al., 2011). The mutants *scp-1* and *scp-2* were identified as carrying mutations in the plant cytochrome P450 monooxygenase gene *CsCYP85A1* and the *de-etiolated-2* gene (*CsDET2*), respectively, which both play important roles in the brassinosteroid (BR) biosynthesis pathway (Hou et al., 2017; Wang et al., 2017). In melon, four recessive dwarfing genes have been reported, *si-1*, *si-2*, *si-3*, and *mdw1,* which were identified from four independent melon lines (Paris et al., 1984; Knavel, 1990; Hwang et al., 2014). Among them, the *si-1* plants exhibit a bush phenotype with an extremely compact growth habit and very short internode lengths (Denna, 1962), and the *si-1* gene is linked to the gene *yv* (*yellow virescent*) (Pitrat, 1991). The internode lengths in *si-2* and *si-3* plants are shorter but the plants are less compact than the *si-1* plants. In *si-2* plants, only the first internodes are short, with no effect on the length of the upper, later-formed internodes, whereas the internode lengths of *si-3* plants are reduced at all plant developmental stages. Of these four melon dwarfing genes, only *mdw1* has been mapped, onto a location between two gene markers in a 1.8 cM region on melon chromosome 7 (Hwang et al., 2014). In pumpkin, a major QTL, *qCmB2*, explained 21.39% of the phenotypic variation for a dwarf bush type and was mapped to a 0.42 Mb region using a high-density genetic map. The *Gibberellin* (*GA*) *20-oxidase* gene was identified as the possible candidate gene controlling vine growth (Zhang et al., 2015). Several dwarfing genes were also identified in watermelon, including two alleles, *dw-1* and *dw-1* *^s^*, and two independent loci, *dw-2* and *dw-3* (Liu and Loy, 1972; Mohr and Sandhu, 1975; Huang et al., 1998). Recently, another dwarf gene, *dsh*, was identified in watermelon from a natural mutation that exhibited both small fruits and short internodes. The *dsh* gene was mapped onto a 27,800 kb long region on watermelon chromosome 7 by BSA-seq analysis and a *gibberellin 20-oxidase-like* gene was predicted as a possible candidate gene (Dong et al., 2018). Although a number of dwarf genes have been reported and mapped in the Cucurbitaceae family, only a few of them have been cloned, and little is known about the underlying molecular mechanisms.

Most of the dwarfing mutations in different crops have been identified as being associated with the loss of function of genes associated with biosynthesis or of response pathways of plant hormones that regulate cell elongation and division (Sasaki et al., 2002; Multani et al., 2003; Nomura et al., 2004; Pearce et al., 2011; Tamiru et al., 2015). Gibberellins (GAs) are well known for playing important roles in controlling plant height, and most GA-deficient or -insensitive mutants are characterized by reduced height and associated developmental phenotypic changes (Sakamoto et al., 2004). Another group of plant hormones, the BRs, have been widely identified as being involved in the regulation of plant height (Hong et al., 2004), and a number of BR-deficient or insensitive dwarf mutants have been isolated in several crops (Tamiru et al., 2015; Hou et al., 2017; Wang et al., 2017). In addition, Multani et al. (2003) identified a different mechanism by which plant height is controlled in dwarf mutants of maize and sorghum and found that the *br2* gene in a maize mutant and the *dw3* gene in a sorghum mutant encoded a protein responsible for the transport of the plant hormones auxins (Multani et al., 2003), indicating that auxins also play a role in the regulation of plant height. Furthermore, more recently identified plant hormones, the strigolactones (SLs), have been revealed as being associated with plant height regulation, controlling stem elongation independently of GAs (de Saint Germain et al., 2013). These findings suggest that the dwarfism trait in different crops may be controlled by various genes that operate *via* different molecular mechanisms and that the classification of dwarfing genes that regulate plant height in combination with desirable pleiotropic effects appropriate for particular crops will enable the breeding of better crop varieties in the near future.

The ATP-binding cassette (ABC) transporter gene family is one of the largest and most ubiquitous gene superfamilies, being present in plants and animals. ABC transporters can transport a wide range of molecules across various membrane types (Do et al., 2018). They are essential for plant development and have been identified as being involved in processes as diverse as gametogenesis, seed development, seed germination, organ formation, and secondary growth (Hwang et al., 2016). The functional ABC transporters are usually composed of two transmembrane domains (TMD) and two nucleotide-binding domains (NBD). Based on the conservation of the NBD sequence, the ABC transporter family can be divided into eight subfamilies (A-H). Of these, several members of the B subfamily (the ABC-B/multidrug resistance/P-glycoprotein, and ABCB/MDR/PGP subfamily), namely ABCB1, ABCB4, ABCB19, ABCB14, and ABCB15, have been identified as auxin transporters involved in plant height regulation (Noh et al., 2001; Kaneda et al., 2011; Kubes et al., 2012). However, the molecular mechanism by which ABCB genes regulate plant height is still unclear, as is whether there is crosstalk between auxins and other hormone pathways in plant height regulation.

In the current study, a dwarf watermelon line, WM102, carrying the single recessive gene, *Cldw-1*, was used to cross with a normal-height watermelon line, WT4. A BSA-seq strategy was used for primary mapping of *Cldw-1* onto the end of watermelon chromosome 9. Based on the whole-genome re-sequencing of the two parental lines, Indel and dCAPS markers were developed from the candidate region and were then used to genotype a large F_2_ mapping population. A mutant ABC transporter gene, *Cla010337*, shown to carry a single nucleotide deletion frameshift mutation in the coding region in the dwarf line, WM102, was identified as the candidate gene for *Cldw-1*.

## Materials and Methods

### Plant Materials and Mapping Populations

WM102 is a dwarf inbred line, which was selected from ‘Bush Sugar Baby’ [accession code: Grif15898; provided by USDA-ARS Germplasm Resources Information Network (GRIN) (https://www.ars-grin.gov/Pages/Collections)] and had been manually self-pollinated for at least four generations. It exhibited short internodes in both the primary and secondary stems. To confirm the inheritance mode and to achieve fine mapping of the *dwarf1* (*Cldw-1*), WM102 was crossed with a normal-height inbred line, WT4, to generate a large F_2_ mapping population. Phenotype scoring for plant height was carried out at 60 days of age, planting in a greenhouse in 2017 and 2018.

To investigate the allelic diversity of the *Cldw-1* gene in natural populations, the *Cldw-1* co-segregating markers dCAPS3 and Indel1 were used to screen a panel of 165 watermelon germplasms of worldwide collection. The plant heights of five plants of each of the 165 watermelon accessions were recorded as normal-height or dwarf plant at 30 and 60 days in the field in 2017 and 2018. Detail information on these accessions is provided in **Table S1**. All the watermelon germplasm and F_2_ population were grown in the greenhouse at the Maozhuang Research Station of Henan Agricultural University (Zhengzhou, China).

### Cytological Characterization of the Dwarf Trait

To compare the cytological characteristics of the internodes between the two parental lines, in autumn 2018, the eighth internodes of three different 45-day-old plants of WM102 and WT4 were separately fixed, washed, dehydrated, dewaxed, embedded, sectioned, and finally viewed using an Olympus-BX53 light microscope, as described previously (Yang et al., 2019). For the measurement of the cell size, three different embedded samples were sectioned and used for investigation. On average, a series of 10 to 20 paraffin sections (10-µm thick) was counted and compared.

In addition to paraffin sectioning, epoxy resin semi-thin sectioning was performed to observe the cell in the eighth internodes between the two parental lines, WM102 and WT4, in autumn 2018. Samples were fixed in 2.5% glutaraldehyde for 4 h at room temperature and rinsed three times with phosphate buffer, post-fixed with 1% osmic acid for 1.5 h at room temperature, washed three times in distilled water, dehydrated in an acetone series, and finally, embedded in LR White acrylic resin. Semi-thin sections were taken and supported on Formvar-coated gold grids and then observed with the Olympus-BX53 light microscope.

### Bulk Pooling and BSA-Seq Analysis

For next-generation sequencing-aided bulked-segregant analysis (BSA-seq), 20 dwarf plants and 20 normal-height plants from the F_2_ mapping populations in the greenhouse were selected at random for DNA extraction. Unexpanded young leaves from these plants were collected into 1.5 mL microcentrifuge tubes, lyophilized in a freeze dryer, and ground into fine powder. Genomic DNA was extracted using the CTAB method (Murray and Thompson, 1980).

The dwarf plant bulk (D-bulk) and the normal-height plant bulk (N-bulk) were generated by pooling equal amounts of DNA from each of the dwarf plants and normal-height plants, respectively. A 5-ug DNA sample was then taken from each of the two bulks and from each of the two parental lines. These samples were used to construct paired-end sequencing libraries, which were sequenced on an Illumina HiSeq™ 2500 platform.

After removing adapter sequences, short reads, and low-quality reads, the clean reads from the two bulks were further rechecked for quality and then used for mapping to the watermelon reference genome 97103 (ftp://cucurbitgenomics.org/pub/cucurbit/genome/watermelon/97103/v1) with a Burrows-Wheeler Aligner (BWA) (Li and Durbin, 2009). GATK (Genome Analysis Toolkit) was used to detect SNPs and small Indels between the two parental lines and the two bulks, respectively (McKenna et al., 2010). SNPs and small Indels from the alignments were called using Samtools, and the output was given in pileup format (Li et al., 2009). The quality score of the SNPs was assigned by Samtools to evaluate the reliability of SNP calling based on the Phred-scaled probability that the consensus is identical to the reference. The reliable SNPs and small Indels were noted and predicted using SnpEff software (Chen et al., 2009), and only the high-quality SNPs, with a minimum of five-sequence read depth were used for BSA-seq analysis.

Two methods were used to detect the genomic regions associated with dwarfism: the Euclidean Distance (ED) algorithm and SNP-Index analysis. The detailed calculation for ED was as described previously (Hil et al., 2013). The ED values were raised to a power of 5 (ED5) to decrease the noise generated by small variations in the estimations. A high ED value suggested that the SNPs in the genomic regions were closely associated with the target genes. The SNP-index is an association analysis method for finding significant differences in genotype frequency between the pools as indicated by ΔSNP-index; the same process as has been detailed previously was followed (Abe et al., 2012; Takagi et al., 2013). The SNP index is calculated as follows: SNP-index (Dwarf) = ρx/(ρX + ρx), SNP-index (WT) = ρx/(ρX + ρx), ΔSNP-index = SNP-index (Dwarf) - SNP-index (WT). Dwarf and WT represent the dwarf plant bulk and the normal-height plant bulk of the filial generation, respectively. ρX and ρx indicate the number of reads of the alleles in the WT and the dwarf parent lines appearing in their pools, respectively. The difference in each locus between the two pools can be observed through the ΔSNP-index. With respect to qualitative characterization, the correlation threshold is the theoretical ΔSNP-index value of the corresponding population, and the correlation threshold of the F_2_ population is 0.67. The regions over the threshold were considered as the associated candidate regions.

### Marker Development and Linkage Analysis

The SSR markers in the candidate region, which were developed from a genome-wide SSR identification (Zhu et al., 2016), were used to screen for polymorphism between the two parental lines. The good polymorphic markers were then used for use in linkage analysis of an F_2_ mapping population containing 124 plants to validate the BSA-seq result. After the initial localization of *Cldw-1* on chromosome 9, a scaffold-based chromosome-walking strategy was undertaken to identify markers that were more closely linked. SNPs and Indels were explored based on the sequence difference between WM102 and WT4 by comparing the genome re-sequencing data. For Indels, only those polymorphisms with ≥3 bp differences were selected for primer design with Primer3 software (http://primer3.ut.ee/). For SNP genotyping, dCAPS markers were developed with dCAPS Finder 2.0 (Neff et al., 1998).

The PCR amplification of molecular markers and subsequent gel electrophoresis were conducted as described by Zhu et al. (2016). Linkage analysis of the *Cldw-1* locus with molecular markers was performed with the Kosambi mapping function in JoinMap 4.0, using the threshold logarithm_10_ of the odds (LOD) score of 5.0.

### RNA Extraction, CDNA Synthesis, and Qrt-PCR Analysis

Total RNA was isolated from different tissues using the Plant RNA kit (Omega, USA) according to the manufacturer’s protocol. The quality and quantity of RNA samples were assessed on a NanoDrop 2000 spectrophotometer (Thermo Scientific, Waltham, MA, USA). cDNA was synthesized using the SuperScript III Reverse Transcriptase (Invitrogen, USA) after the elimination of genomic DNA from the RNA. The expression pattern of the candidate gene was examined by performing qRT-PCR analysis of several tissues from WT4 and WM102, including the hypocotyl and the entire root system, the first leaf near the apical meristems, and the entire stems of 35-day-old plants. All experiments were performed with three biological and four technical replicates. The *ClCAC* (*Clathrin adaptor complex subunit*, *Cla020794*) gene, which has been validated with respect to stable expression in different watermelon organs and tissues under various conditions (Kong et al., 2014), was used as an internal control. qRT-PCR was performed using the SYBR Green PCR Master Mix (Applied Biosystems Inc., USA) in an iCycleriQTM 5 Multicolor Real-Time PCR detection system (Bio-Rad, USA). The threshold cycle (Ct) value of the *CAC* gene was subtracted from that of *Cldw-1* to obtain a ΔCt value. The Ct value of the control sample, WM102, was subtracted from the ΔCt value to obtain a ΔΔCt value. The fold changes in expression level relative to WM102 were expressed as 2^-ΔΔCt^. The *ClDW1*-specific qRTPCR primers were obtained using Primer3web (http://primer3.ut.ee/). We analyzed the primer specificity by comparing it with the watermelon genomic sequence. Gene-specific primers of *Cldw-1* and *ClCAC* are provided in **Table S2**. *Cldw-1* gene-specific qRT-PCR primers were developed, locating on the 11^th^ exons, with a product size of 83 bp. The thermal cycler settings for qRT-PCR consisted of an initial hold at 95°C for 2 mins, 40 cycles of 95°C for 15 s and 60°C for 30 s, then 95°C for 15s, 60°C for 1 min, and 95°C for 15s.

### Phylogenetic Analysis of CIDW-1

To investigate the phylogenetic relationships of the watermelon ClDW-1 protein with members of the identified subfamily B of the ABC transporter superfamily, all 20 members of the ABCB subfamily in the *Arabidopsis* genome (https://www.arabidopsis.org/), with another two ABCB proteins, ZmBR2 (GenBank accession number: AY366085) and SbDW3 (GenBank accession number: AY372819) (Multani et al., 2003), were used in this study. The full-length protein sequences of all the subfamily B members were aligned using the CLUSTX program (Chenna et al., 2003), and the alignment report was visualized by Gendoc. The phylogenetic tree was constructed using the maximum likelihood method in MEGA 5 with a bootstrap of 1,000 replicates (Tamura et al., 2011).

### Statistical Analysis

The χ^2^-test for goodness-of-fit was used to test for deviation of the observed data from the theoretically expected segregation for the dwarf plants in the F_2_ population from the WM102 × WT4 cross. Student’s *t*-test was used for comparison of fold change expression levels between WM102 and WT4. A difference was considered to be statistically significant when *P* < 0.05. Summary statistics are presented as mean ± standard deviation (SD).

## Results

### Morphological Characterization and Inheritance of Dwarf Trait in Watermelon

**Figure 1:**
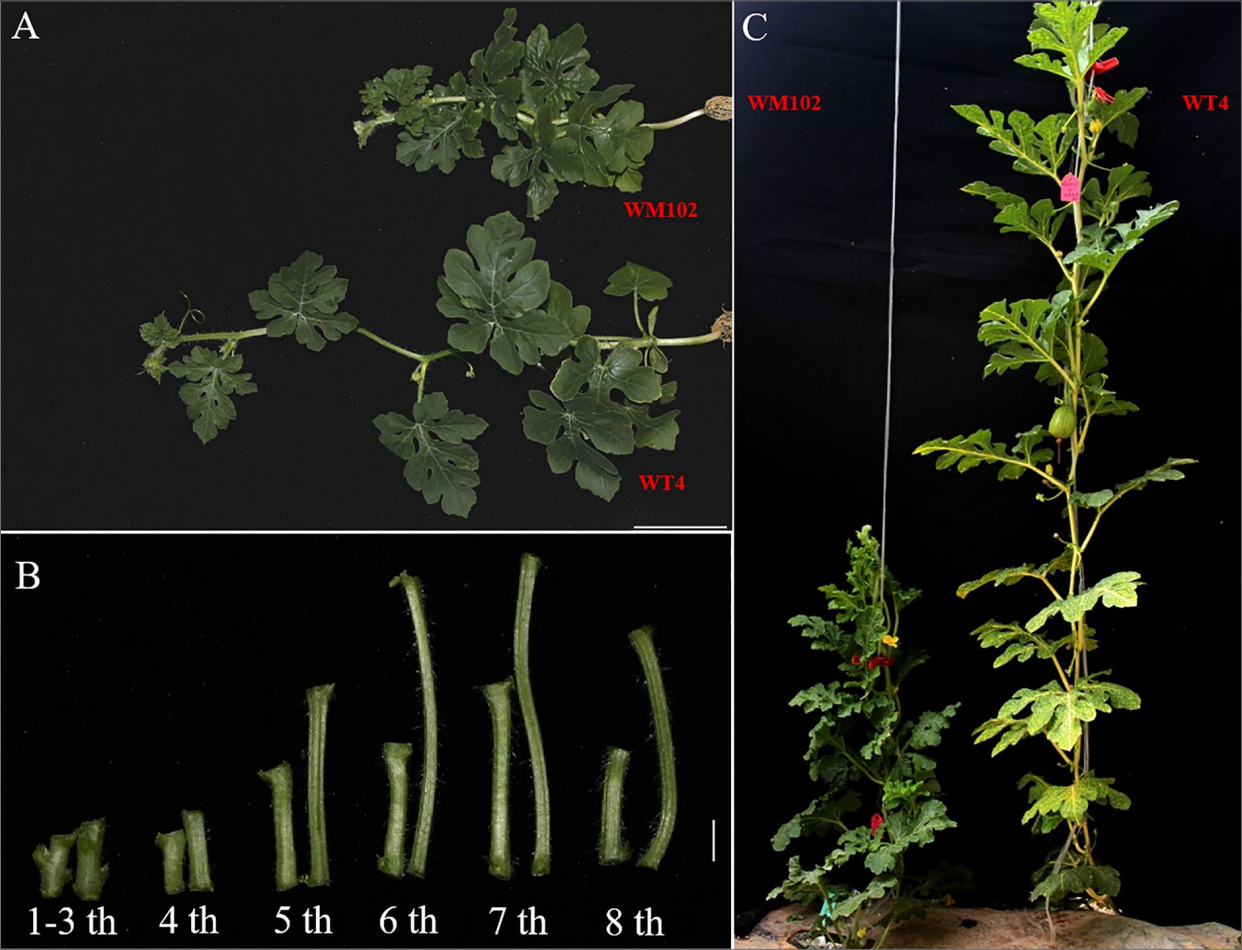
Morphological characterization of two parental lines, WM102 and WT4. **(A)** 35-day-old plants of WM102 and WT4 grown in the growth chamber. Bar = 5 cm. **(B)** From left to right, the first three internodes and then the 4^th^ to 8^th^ internodes of WM102 and WT4. In each group, the left represents inbred line WM102, and the right represents inbred line WT4. Bar = 1 cm. **(C)** 45-day-old plants of WM102 and WT4 grown in the field.

**Figure 2:**
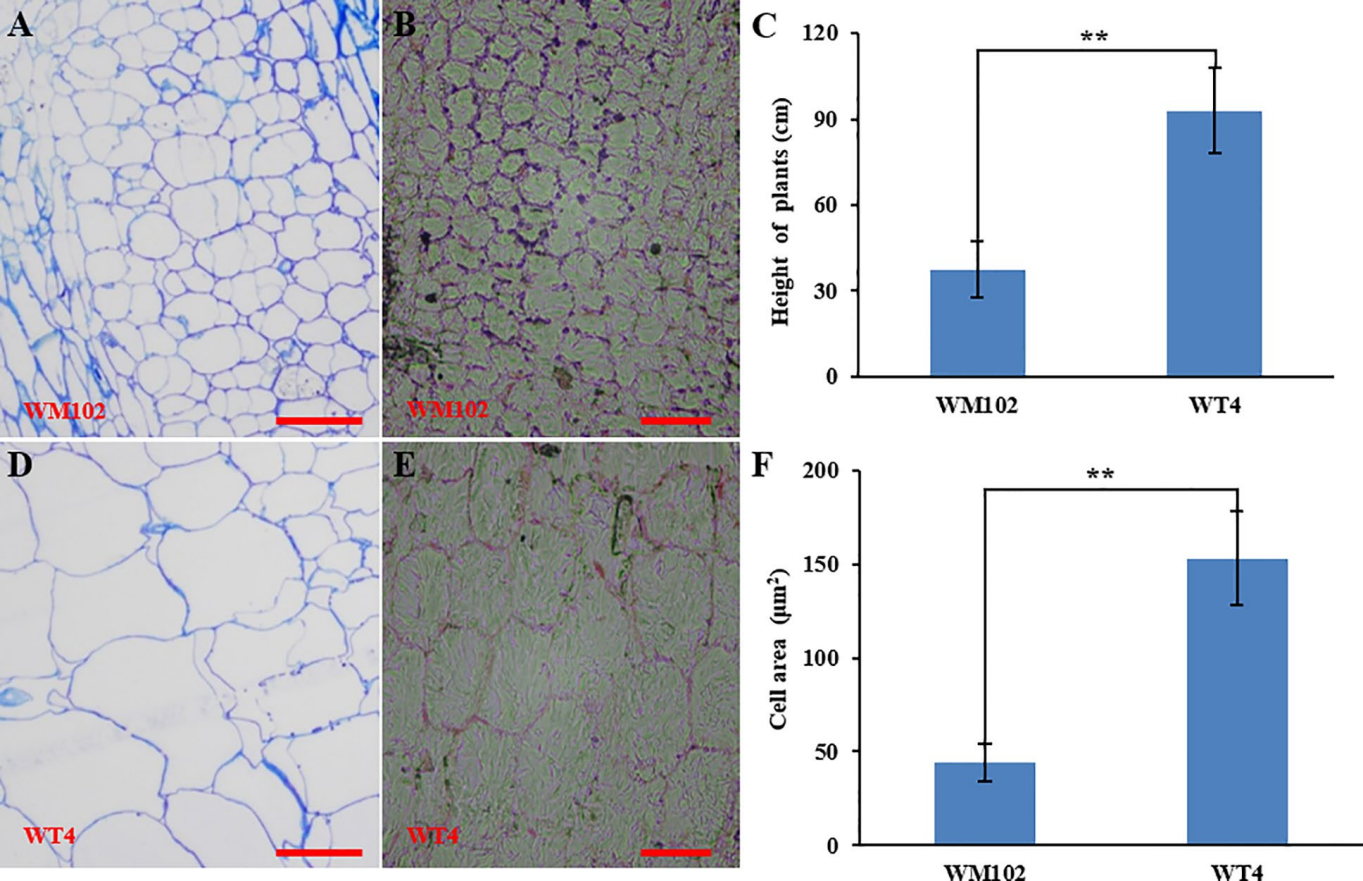
Cytological characterization of the parental lines, WM102 and WT4. **(A**, **B)** Paraffin sections and semithin sections of the eighth internodes in 45-day-old plants of WM102. **(C)** Quantification of the plant height of 45-day-old plants of WM102 and WT4 in autumn 2018. Asterisks indicate that the WM102 plants are significantly shorter than WT4 plants (**P < 0.01). **(D**, **E)** Paraffin sections and semithin sections for the eighth internodes in 45-day-old plants of WT4. **(F)** Quantifications of cell size of the eighth internodes in three different WM102 and WT4 plants in autumn 2018. Asterisks indicate that the cell size in the WM102 plants is significantly smaller than that in WT4 (**P < 0.01). Scale bars: 100 μm.

Comparative morphological characterization between the two parental lines was conducted by measuring the hypocotyl length, main stem length, and internode length and comparing their values at different developmental stages in spring and autumn 2018. The hypocotyl length of 15-day-old seedings in WM102 was about 1.52 cm, which was almost half of that in WT4 (**Table 1** and **Figure S1**). Though the plant height and internode length were varied at different developmental stages in various environments, the relative length of plant height and internode length were significantly reduced in the dwarf inbred line, WM102, being almost half that in the normal-height line, WT4 (**Table 1**, **Figure 1** and **Figure S1**). However, there were no significant differences between the two parental lines in terms of the length of the first three nodes, which were very short and clustered together in both accessions. The difference in internode length and plant height between the two parental lines became more evident from the fourth node (**Figures 1A, B**). In addition, the lateral internode and tendril lengths were also significantly shorter in the dwarf line WM102. To further compare the cell structure of the internodes of WM102 and WT4 plants, the cell sizes of the eighth internodes of 45-day-old plants were viewed under a light microscope by semi-thin sectioning and paraffin sectioning. The cytological characterization showed that the cells, especially the parenchyma cells, in the internodes of WM102 plants were significantly smaller than in those of WT4 (**Figure 2**), a finding consistent with the shorter internode.

**Table 1:** Morphological characterization of two parental lines in spring 2018.

Trait	Stage	WT4	WM102
Hypocotyl length (cm)	15-day-old	3.42 ± 0.59	1.52 ± 0.48
Plant height (cm)	30-day-old	185.20 ± 10.52	86.80 ± 7.43
	60-day-old	235.30 ± 6.26	128.72 ± 8.1
Internode length (cm)	30-day-old	5.54 ± 0.11	3.27 ± 0.31
	60-day-old	6.36 ± 0.11	4.16 ± 0.22

The phenotype of plant height for the F_2_ population was investigated in spring and autumn 2018 (named 2018S-F2 and 2018A-F2, respectively), and genetic analysis revealed that the segregation of normal-height and dwarf plants was consistent with the segregation of a single recessive gene in both 2018S-F2 and 2018A-F2, which was named as *Cldw-1* (**Table 2**).

**Table 2:** Segregation analysis of the dwarf plant in the F_2_ population.

Population	Total	Dwarf type	Normal type	Expected ratio	χ^2^	*P*
F_1_	15	0	15	–	–	–
2018S-F_2_	538	140	398	3:1	0.2478	>0.05
2018A-F_2_	515	144	371	3:1	2.2531	>0.05

We selected 20 dwarf plants and 20 normal-height plants at random from the F_2_ population for pooling into two bulks: the dwarf plant bulk (D-bulk) and the normal-height plant bulk (N-bulk). The DNA from each of the two bulks and each of the two parental lines was used for paired-end library construction and next-generation sequencing (NGS). After filtering the raw reads, the two re-sequencing parental lines generated 79,907,647 and 73,483,217 clean reads totaling 23.94 and 22.02 Gb for WT4 and WM102, respectively. In addition, a total of 33.13 Gb clean reads were obtained (15.39 Gb from the D-bulk and 17.74 Gb from the N-bulk) with an average 34 × depth of the draft watermelon reference genome. All the clean reads of the two parental lines and the two bulks were then mapped to watermelon reference genome 97103, with more than 80% of the total reads being precisely mapped onto specific watermelon chromosomes. In total, 401,140 SNPs were detected after removing SNPs with low coverage or discrepancy between the parental lines and the bulks, and only 115,867 high-quality SNPs were finally retained.

**Figure 3:**
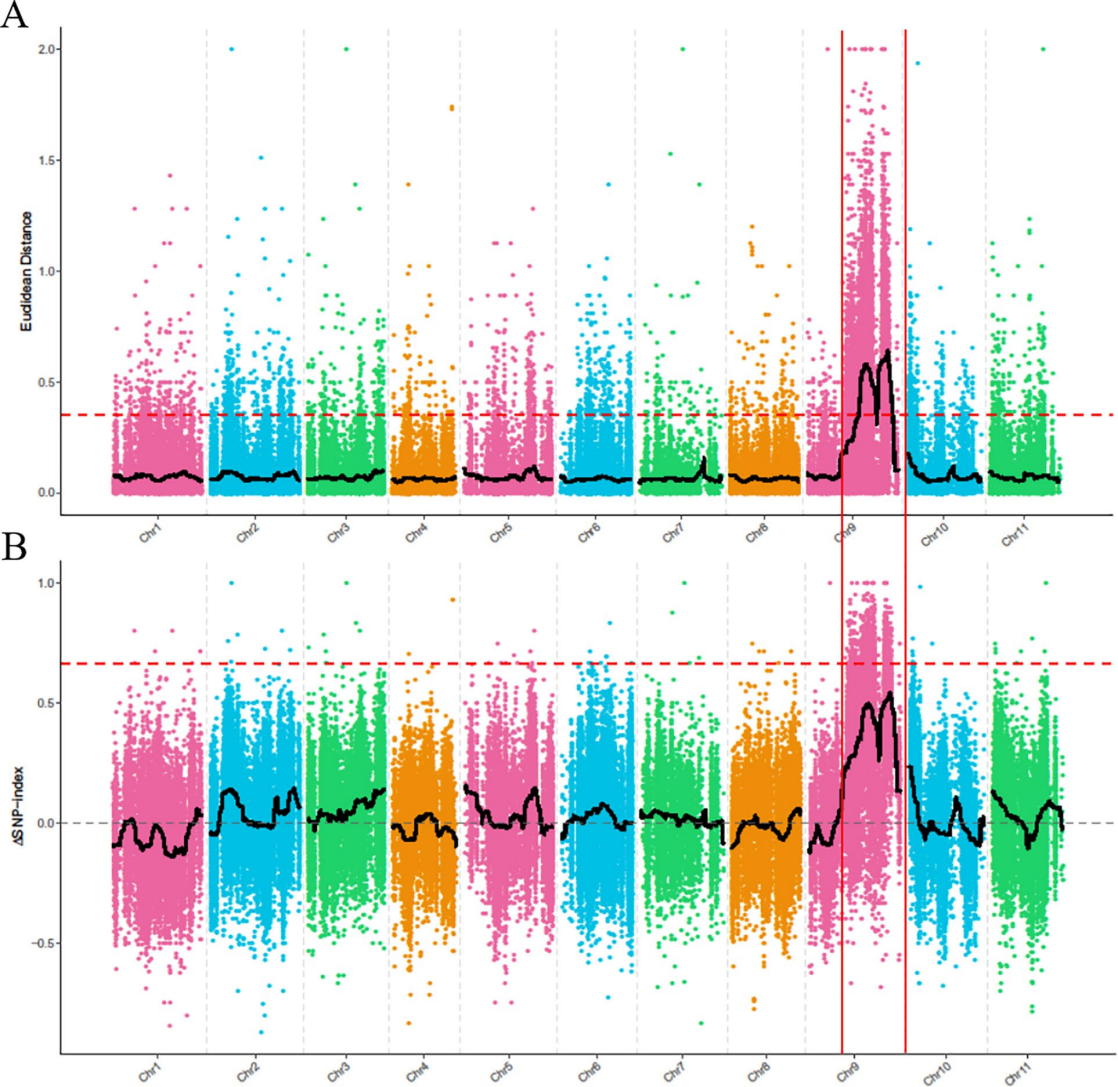
BSA-seq analysis using Euclidean Distance (ED) algorithm **(A)** and SNP-Index method **(B)**. The colored dots represent the calculated ED value **(A)** and ΔSNP-index value **(B)** of each SNP locus, and the red dashed line represents the significant threshold.

To identify the genomic region associated with the dwarf phenotype, we first used the ED algorithm to measure the allelic segregation of SNPs between the D-Bulk and the N-Bulk. There were two statistically significant peaks of ED detected on chromosome 9, which were located close together in a large region between 19,710,000 and 26,970,000 bp (**Figure 3A**). Then, the SNP-Index of each SNP locus was also calculated between the two bulks using the high-quality SNPs, but no significant region was identified to be associated with the dwarf phenotype. However, two obvious adjacent peaks were still detected under the significant threshold, and these were located in the same candidate regions as had been detected by ED analysis (**Figure 3B**). This indicated that the two candidate regions probably contained the candidate gene regulating plant height in watermelon. One peak was located between 22,540,000 and 22,810,000 bp, and the other peak was located from 28,640,000 to 31,590,000 bp on chromosome 9.

### Validation of the BSA-Seq Result and Fine Mapping of the *Cldw-1* Gene

**Figure 4:**
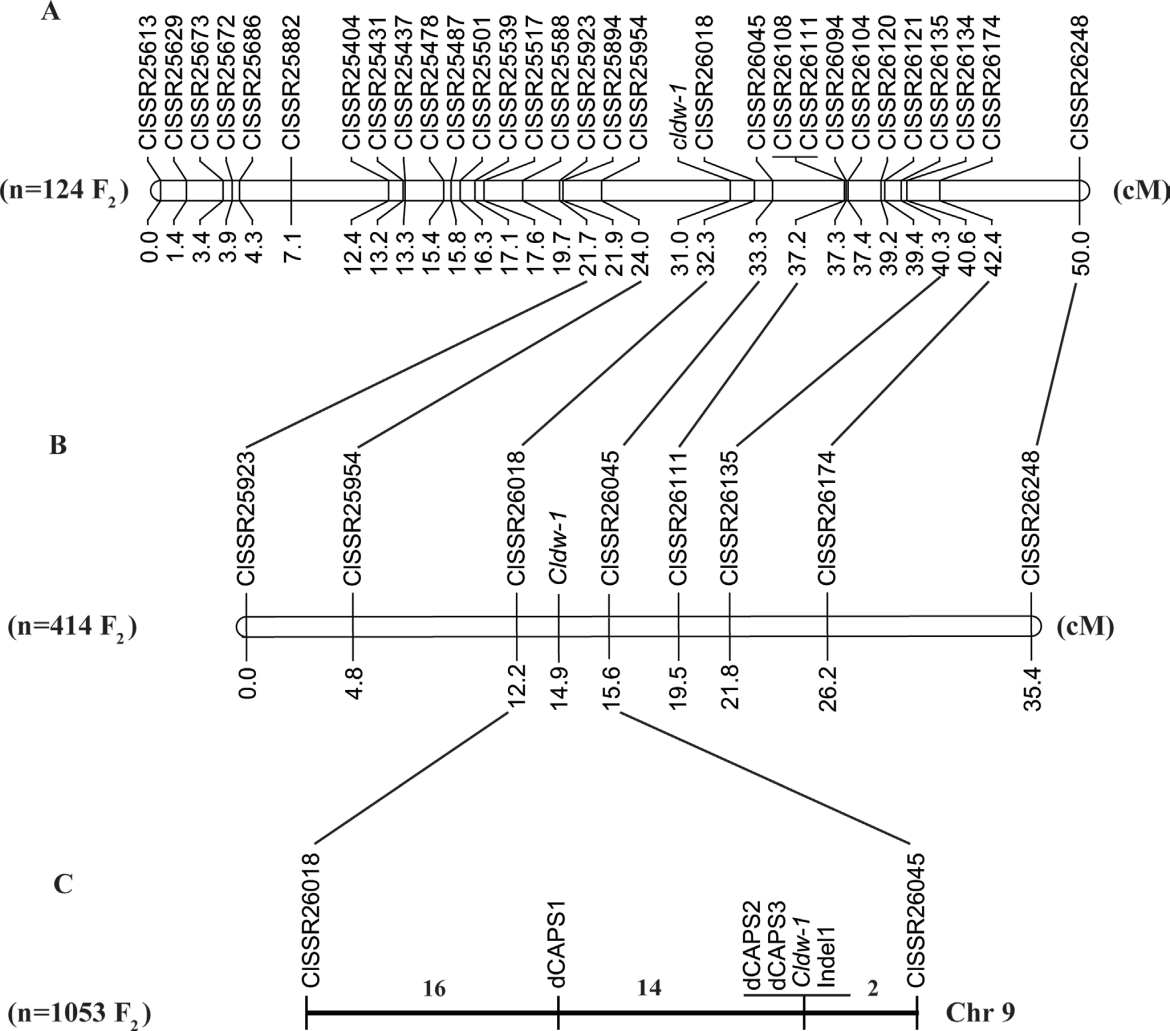
Fine mapping of *Cldw-1* in watermelon. **(A)** Validation of the BSA-seq result by linkage analysis of the *Cldw-1* gene with SSR markers in 124 F_2_ plants. **(B)** Fine mapping of the *Cldw-1* gene using eight closely flanked SSR markers in 414 F_2_ plants. **(C)** The *Cldw-1* gene was finally mapped between marker dCAPS1 and ClSSR26045 in 1,053 F_2_ plants. The numbers above chromosomes represented the recombinants between adjacent markers in the 1,053 F_2_ plants.

A total of 52 SSR markers in the candidate regions on chromosome 9 were selected from a genome-wide SSR marker identification in watermelon (Zhu et al., 2016) for screening polymorphisms, of which 22 showed clear bands and polymorphism between WT4 and WM102. A further 18 SSR markers were selected from these for genotyping a small F_2_ population of 124 plants, and the *Cldw-1* gene was mapped between ClSSR25954 and ClSSR26018 within a genetic distance of 8.5 cM, indicating that the BSA-seq mapping result was consistent with the linkage analysis. To further shorten the genetic distance of markers flanking the target gene, a chromosome-walking strategy was used, and additional SSR markers were selected from the candidate region for polymorphism screening. After three rounds of chromosome walking, 30 SSR markers were used for the final map construction, and the *Cldw-1* gene was still mapped between ClSSR25954 and ClSSR26018 within a genetic distance of 8.3cM (**Figure 4A**). Detail information on these SSR markers is provided in **Table S2**.

To check the primary mapping results, we selected an additional 414 F_2_ plants, using eight SSR markers closely linked to *Cldw-1* for genotyping. We found that the two closely flanking markers of *Cldw-1* were slightly different in this larger population, with the *Cldw-1* gene being flanked by ClSSR26018 and ClSSR26045 with map distances from the target gene of 2.7 and 0.7 cM (**Figure 4B**), respectively. This discrepancy between the results from the two different mapping populations was probably because the primary mapping of *Cldw-1* used a small mapping population and a high density of markers, which led to the marker order not precisely reflecting their genetic positions. The latter two markers, ClSSR26018 and ClSSR26045, identified from the larger mapping population, were located on the same scaffold with a physical interval of 170.05 kb. For fine mapping of the *Cldw-1* gene, these two closely linked markers (ClSSR26018 and ClSSR26045) were used to genotype an extended, even larger F_2_ mapping population of 1053 plants. Additionally, the whole genome re-sequences of the two parental lines were aligned to the watermelon reference genome, and SNPs and Indels were identified in this candidate region for marker development. Three dCAPS markers and one Indel marker were developed for genotyping the recombinant plants. Finally, the *Cldw-1* gene was mapped between dCAPS1 and ClSSR26045, which had 14 and 2 recombinants, respectively. They were physically located in a 107 kb region from 30,394,615 to 30,501,700 kb on chromosome 9. Of the new mapped markers, dCAPS2, dCAPS3, and Indel1 were all co-segregated with the *Cldw-1* locus (**Figure 4C**).

**Figure 5:**
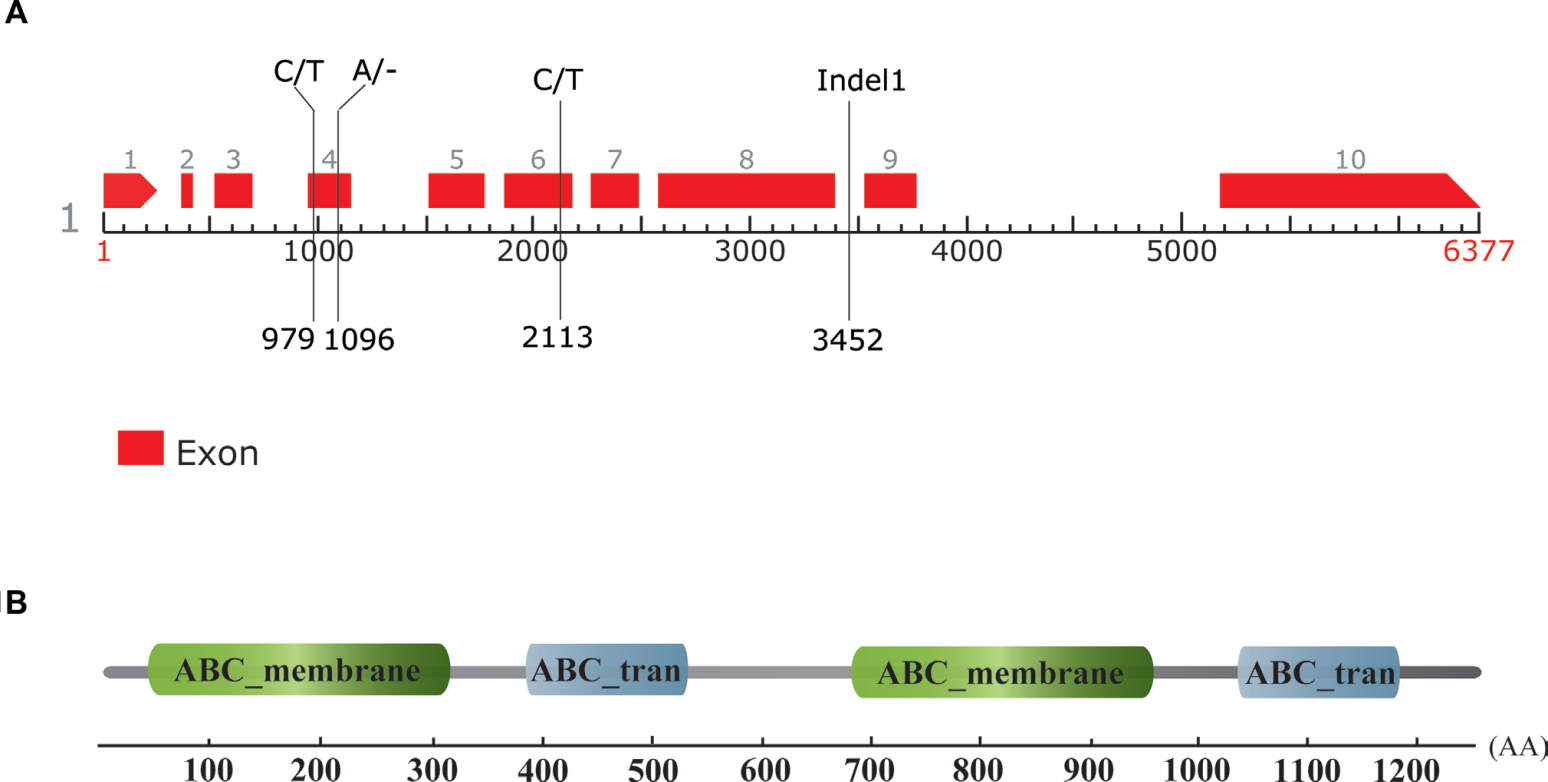
Gene prediction, sequence comparison, and motif identification of the Cldw-1 gene in watermelon. **(A)** The genomic structure of the *Cldw-1* gene in watermelon. Two SNPs and one nucleotide deletion in the exons and a 5-bp deletion in the intron (marker Indel1) were detected between the two parental lines; their physical positions in the genomic sequence are indicated. The left nucleotides and the right nucleotides at each site represent the normal-height line, WT4, and the dwarf line, WM102, respectively. **(B)** The *Cldw-1* gene was annotated to encode four conserved domains in the deduced amino acid sequence.

According to the annotation of the watermelon reference genome 97103, there were six genes predicted to be in the 107 kb candidate region, namely *Cla010332*, *Cla010333*, *Cla010334*, *Cla010335*, *Cla010336*, and *Cla010337*. The physical positions and gene annotations of these genes are provided in **Table S3**. The SNPs and Indels between the two parental lines were checked with reference to these genes, and no difference was detected for five of the six genes (*Cla010332* to *Cla010336*). For *Cla010337*, three SNPs and two Indels were detected between the two parental lines. For the three SNPs, one SNP was located in the intron, and two SNPs were located in the exons (**Figure 5**). Of the two Indels, a 1-bp deletion was present in the 4^th^ exon of the dwarf line, WM102, with another 5-bp deletion being detected in the 8^th^ intron of WM102, and two markers, dCAPS3 and Indel1, were developed from these two Indels, respectively. Both of them showed co-segregation with the dwarf trait in the F_2_ population, indicating that *Cla010337* was the candidate gene for *Cldw-1*.

**Figure 6:**
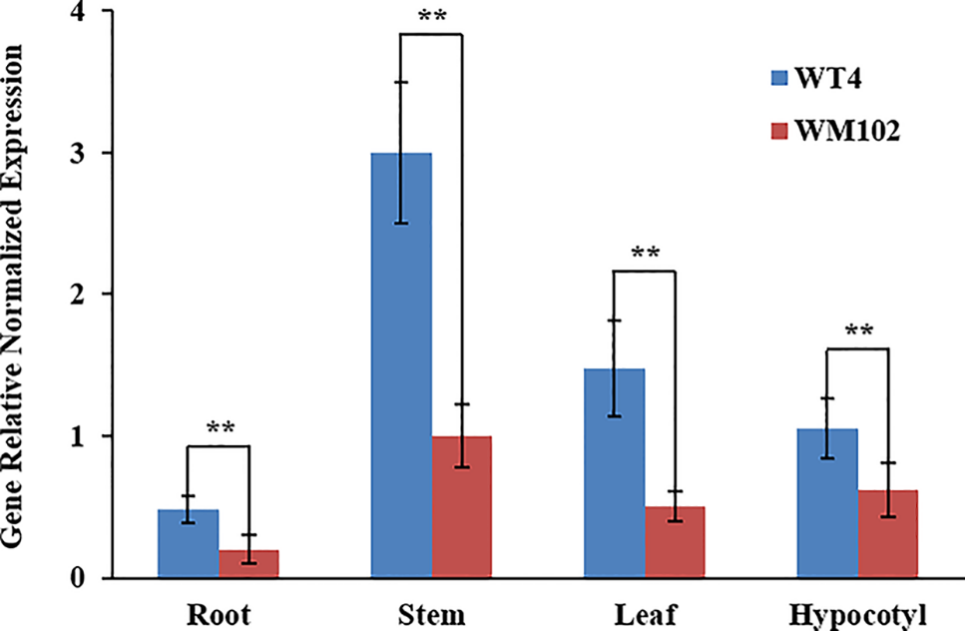
Expression analysis of the *Cldw-1* gene in different tissues between WM102 and WT4 using qRT-PCR. Expression of *Cldw-1* was decreased in all WM102 tissues. Error bars represent the SE. Significant differences were determined according to Student’s t-test (**P < 0.01).

We compared the expression levels of *Cla01033*7 in the root, hypocotyl, leaf, and stem tissues of WT4 and WM102 using qRT-PCR. The results showed that *Cla010337* was expressed in all the tissues tested, with the highest expression level being in the stem (**Figure 6**). The expression levels in each of the test tissues from the dwarf line, WM102, were significantly lower than those from the normal-height line, WT4, suggesting that the 1-bp deletion frameshift mutation in the 4^th^ exon greatly affected the expression and function of *Cla01033*7.

### Gene Annotation, Sequence Alignment, and Allelic Diversity of *Cldw-1*

**Figure 7:**
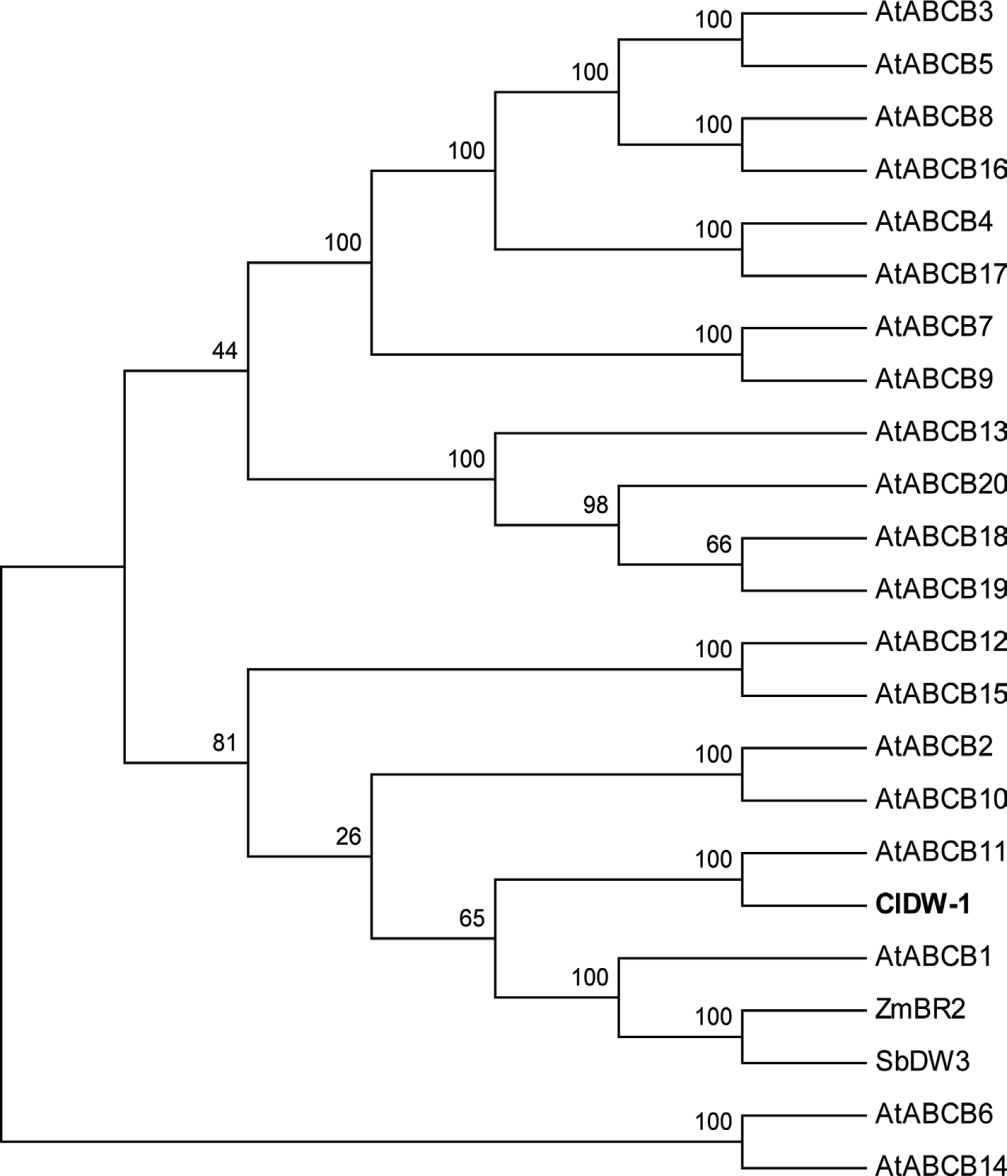
Phylogenetic analysis of proteins of ABC transporter subfamily B. The proteins used for phylogenetic analysis including 20 genes from *Arabidopsis* (AtABCB1-19), one from maize (ZmBR2), one from sorghum (SbDW3), and one from watermelon (ClDW-1).

The gene size of *Cla010337* in the watermelon reference genome 97103 was 6,377 bp, which was predicted to contain nine introns (**Figure S2**), and the full length of the coding sequence (CDS) was 3,753 bp, encoding a protein of 1,250 amino acids. The single nucleotide deletion of the 631^th^ in the CDS resulted in a frameshift mutation in the dwarf line, WM102, which led to a truncated protein (**Figure 5A**). Gene prediction and function annotation revealed that *Cla010337* encoded an ABC transporter that belongs to the ATP-binding cassette transporter superfamily. The deduced amino acid sequence of Cla010337 contained two conserved ABC_membrane domains and two conserved ATP-binding domains (**Figure 5B**, **Figure S3**). The ABC transporter superfamily is divided into eight subfamilies (A-H), with Cla010337 being a member of the B subfamily. We further constructed a phylogenetic tree using ClDW-1 and other subfamily B proteins, including all the ABCB subfamily members of *Arabidopsis*, and another two ABCB proteins, ZmBR2 and SbDW3, which regulated the height of dwarf plants in maize and sorghum, respectively (Multani et al., 2003; Ye et al., 2013). The phylogenetic analysis revealed that ClDW-1 showed a close relationship with a group of well-known proteins regulating plant height, comprising AtABCB1, ZmBR2, and SbDW3 (**Figure 7**). This suggested that Cldw-1 in watermelon may have similar functions in regulating plant height development.

The allelic diversity of the *Cldw-1* gene in natural watermelon germplasm was examined by observing the plant height of other 165 watermelon accessions (**Table S1**) and through scoring in 2017 and 2018 field trials; normal plant height was exhibited in all cases. Both the Indel1 and dCAPS3 markers were then used for genotyping this worldwide collection of accessions. The results showed that only the dCAPS3 marker was completely consistent with the phenotyping (**Figure S4A**), while the Indel1 marker developed from the 8^th^ intron was not in complete agreement with the phenotype (**Figure S4B**), suggesting that the 5-bp deletion in the intron of *Cla010337* was not conserved among different watermelon accessions.

## Discussion

Plant height is an important trait that affects crop architecture, resistance to lodging, fruit yield, and mechanical harvesting. The two main cultivation methods for watermelon in China are in the open field (creeping habit) and in protected cultivation (hanging vine habit). Most of the commercial cultivars of watermelon have tall stems with long branches, and a lot of time and labor is expected to carry out pruning. A dwarf watermelon plant with short internodes of the main stem and branches can increase the plant density and hence enhance yield per unit land area and reduce labor requirements for pruning. As a consequence, the development of dwarf or compact watermelon cultivars has become one of the main targets in watermelon breeding.

Dwarf genetic resources are important for genetic improvement and crop breeding for altered plant architecture, and at least four recessive dwarf genes have been identified in watermelon (Guner and Wehner, 2004). The WM102 used in this study was developed from ‘Bush Sugar Baby,’ carrying the *dw-1* gene (Mohr, 1956), and it had short internodes with two to three branches. Cytological comparison between normal and dwarf plants in the present study revealed that cell size was significantly smaller in WM102, suggesting that the loss-of-function of *Cldw-1* led to the short cells and internodes (**Figure 2**). *dw-1* *^s^* is allelic to the *dw-1* gene, with plants expressing *dw-1* *^s^* having vine lengths intermediate between normal height and dwarf (Dyutin and Afanas’eva, 1987). The *dw-2* plants have a main stem length of approximately 100 cm and internode lengths of approximately 5 cm, with 5-11 branches (Robinson et al., 1976), while plants with *dw-3* have dwarf stems with fewer, lobed leaves (Huang et al., 1998). A dwarf plant was recently identified with small fruit, and genetic analysis revealed that it was controlled by a recessive gene, *dsh* (Li et al., 2016). Though some of these dwarfing genes in watermelon have been known for a long time, none of them have been cloned, and how they control plant height is still unknown.

Compared with the reverse-genetics approach in gene cloning, map-based cloning is labor-intensive and time-consuming, which restricts its use in gene identification in horticultural crops. However, forward genetics relying on random mutagenesis can precisely identify a novel gene that acts in a particular pathway, revealing novel functions for known genes. This can provide a true understanding of gene function and the mechanism by which gene networks regulate a target trait (Gillmor et al., 2016; Yang et al., 2018). In comparison with model species, only a few genes have been identified by map-based cloning in watermelon. With the high efficiency and cost-effectiveness of next-generation sequencing technology, BSA-seq can quickly detect thousands of markers across the genome with adequate coverage and identify the candidate regions associated with the target trait. This approach has been used not only in gene/QTL mapping (Lu et al., 2014; Song et al., 2017; Knorst et al., 2019; Liu et al., 2019) but also for sequencing and genome-wide association studies (Turner et al., 2010; Yang et al., 2015; Zou et al., 2016). The power of BSA-seq is affected by the size of the bulked sample of extreme individuals, sequencing strategy and depth, and the genetic architecture of the target trait (Zou et al., 2016). In the present study, we used BSA-seq for primary mapping by bulk samples of 20 dwarf plants and 20 normal height plants with genome coverage of 34 × for each bulk, and two regions on chromosome 9 were identified as candidate intervals for the *Cldw-1* gene (**Figure 3**). The identified candidate regions were discontinuous and large, which probably resulted from the relatively small sample size and the lower coverage of each bulk. However, it still provided the correct direction for further fine mapping of the *Cldw-1* gene by linkage analysis, and it was efficient in the initial mapping of a target trait. Furthermore, the marker dCAP3 developed in this study co-segregated with the dwarf trait in the F_2_ mapping population and the natural population (**Figure S4**), indicating that the functional dCAP3 marker of the *Cldw-1* gene could be used in marker-assisted selection (MAS) for plant height breeding or as the basis for genome selection breeding in watermelon.

Plant hormones play central roles in the regulation of plant height by coordinating cell elongation and division, and the dwarf plant trait is controlled by genes that are involved in a complex regulatory network responsible for the biosynthesis or signal transduction of gibberellins (GAs) and brassinosteroids (BRs) (Liu et al., 2018). In the Cucurbitaceae family, several dwarf or compact habit genes associated with the GA or BR pathway have been mapped or cloned. In cucumber, the dwarf or compact genes *scp-1* and *scp-2* have been shown to be members of the plant cytochrome P450 monooxygenase gene *CsCYP85A1* and *CsDET2*, and both have been shown to have functions in the BR biosynthesis pathway (Hou et al., 2017; Wang et al., 2017). The *gibberellin* (*GA*) *20-oxidase-like* gene was identified as the candidate gene for the *dsh* gene controlling the dwarf phenotype in watermelon (Dong et al., 2018) and a major QTL for dwarf bush type in pumpkin (Zhang et al., 2015). Furthermore, GA-response or -biosynthesis mutants have been extensively used to reduce plant height in wheat, maize, and rice. Except for GAs and BRs, dwarfing genes involved in the auxin pathways have also been discovered. Auxins have been identified as being involved in modulating plant height in maize, sorghum, and *Arabidopsis* by creating a gradient regulated *via* its transporters, such as members of the ABCB subfamily (Noh et al., 2001; Multani et al., 2003; Ye et al., 2013). The dwarfing mutations in maize (*ZmBR2*) and sorghum (*SbDW3*) reduced plant height by compacting the lower internodes without any adverse effect on the remainder of the plant (Multani et al., 2003). In *Arabidopsis*, the mutant of its ortholog, *AtPGP1-2*, and the *atabcb1 atabcb19* double knockout exhibited reduced plant height (Noh et al., 2001; Ye et al., 2013). The *Cldw-1* dwarfing gene identified in the current study also encoded an ABC transporter of the ABCB subfamily and clustered with AtABCB1, ZmBR2, and SbDW3 in the phylogenetic analysis, suggesting that it may have a conserved function in regulating plant height in both monocots and dicots. Future research will focus on the function and regulatory network of the *Cldw-1* gene, which will be helpful for elucidating the molecular mechanism of plant height regulation in watermelon.

## Data Availability Statement

GenBank accession for the re-sequencing dataset of WT4 and WM102 (PRJNA551784).

## Author Contributions

MZ, SS, JL, HL, SY, HY, and KZ performed phenotyping in F_2_ plants and fine mapping. LY, HZ, DL, and JH contributed to data processing and analysis. SY contributed to microscopic analysis. LY and HZ wrote the manuscript. All authors reviewed and approved this manuscript.

## Funding

This work was supported by grants from the National Natural Science Foundation of China (31872133 and 31902041), the Key Scientific and Technological Research Projects of Henan Province (No. 192102110042), and the Project for Scientific and Technological Activities of Overseas Students of Henan Province.

## Conflict of Interest

The authors declare that the research was conducted in the absence of any commercial or financial relationships that could be construed as a potential conflict of interest.

## Supplementary Material

Table S1 The list of watermelon germplasm used for analyzing allelic diversity of Cldw-1 gene.

Table S2 The list of primers used for mapping and qRT-PCR analysis.

Table S3 The physical position and gene annotation for the six genes in the candidate region.

Figure S1 Comparison of hypocotyl length, plant height and internode length between two parental lines. The plants measured were grown in the greenhouse in 2018 Spring. **(A)** The hypocotyl length of 15-day-old seeding. The plant height **(B)** and average internode length © of 30-day-old and 60-day-old plants.

Figure S2 The genomic sequence and exon-intron structure of ClDW-1 gene. The highlight sequences were indicated as exons.

Figure S3 Sequence alignment of ClDW-1 with its homologs in other species for ABC_membrane domain and ATP-binding domain. The proteins used for sequence alignment including 20 genes from *Arabidopsis* (AtABCB1-19), one from maize (ZmBR2), one from sorghum (SbDW3), and one from watermelon (ClDW-1).

Figure S4 The allelic diversity of Cldw-1 gene in different watermelon germplasms using marker dCAPS3 **(A)** and Indel1 **(B)**. M represented marker (100bp DNA ladder), P1 represented the normal height line WT4, P2 represented the dwarf line WM102, the number from 1 to 165 was corresponded to the natural watermelon germplasms in accordance with the code in **Table S1**.
