# Supplementary figures and images for: A Single Nucleotide Deletion in an ABC Transporter Gene Leads to a Dwarf Phenotype in Watermelon
Source: Front Plant Sci. 2019 Nov 13;10:1399. doi: 10.3389/fpls.2019.01399 (PMC6863960; doi:10.3389/fpls.2019.01399)

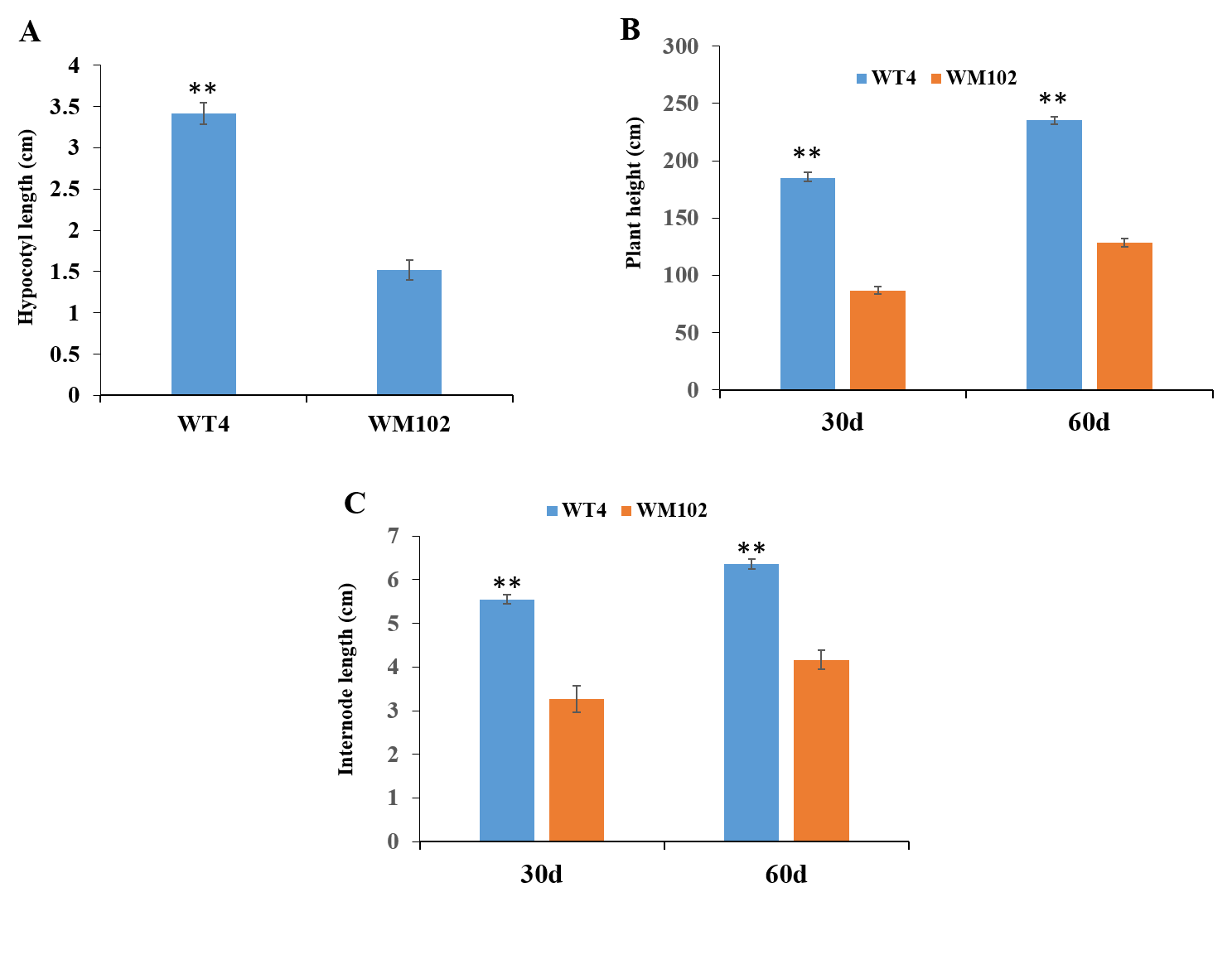

Supplement: Figure S1 — Comparison of hypocotyl length, plant height and internode length between two parental lines. The plants measured were grown in the greenhouse in 2018 Spring. (A) The hypocotyl length of 15-day-old seeding. The plant height (B) and average internode length © of 30-day-old and 60-day-old plants. [file Image_1.tif]

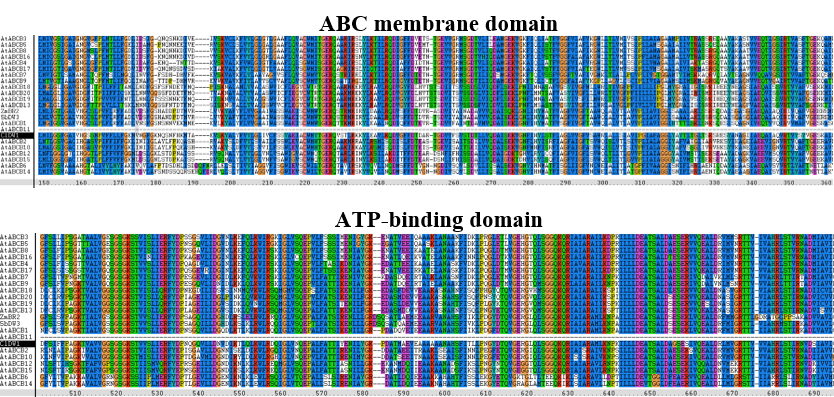

Supplement: Figure S3 — Sequence alignment of ClDW-1 with its homologs in other species for ABC_membrane domain and ATP-binding domain. The proteins used for sequence alignment including 20 genes from Arabidopsis (AtABCB1-19), one from maize (ZmBR2), one from sorghum (SbDW3), and one from watermelon (ClDW-1). [file Image_3.tif]

**A**

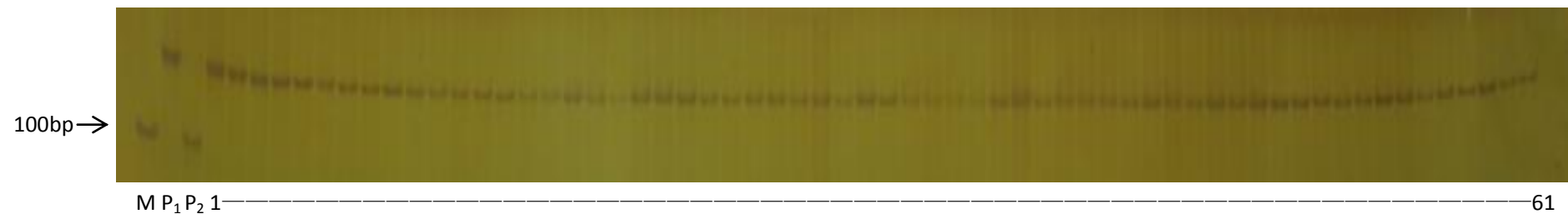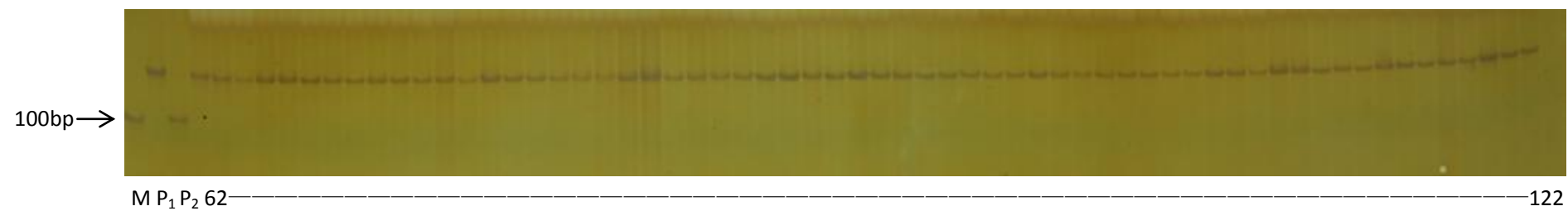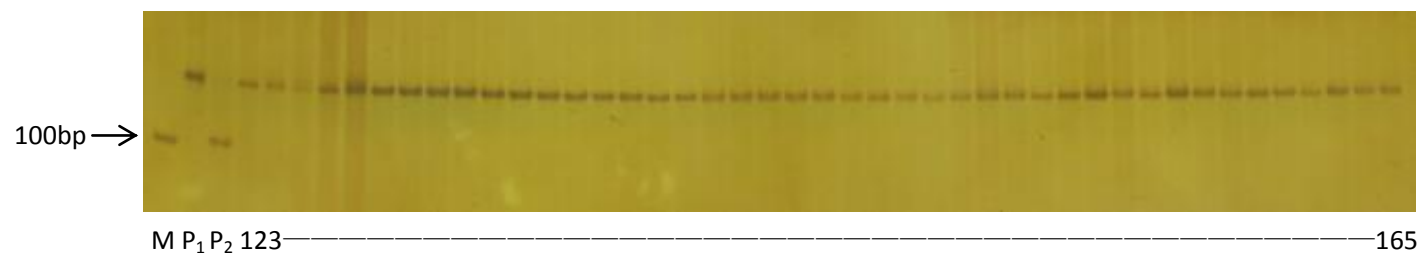

**B**

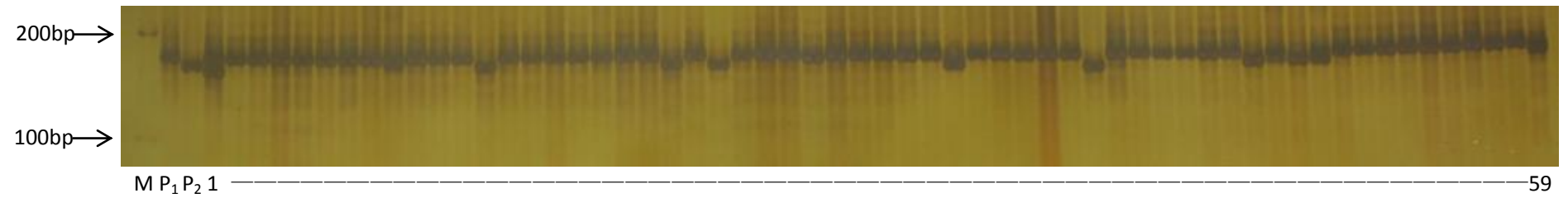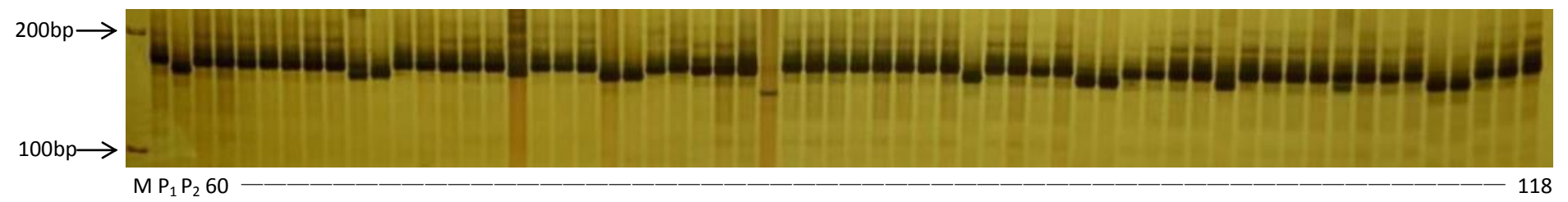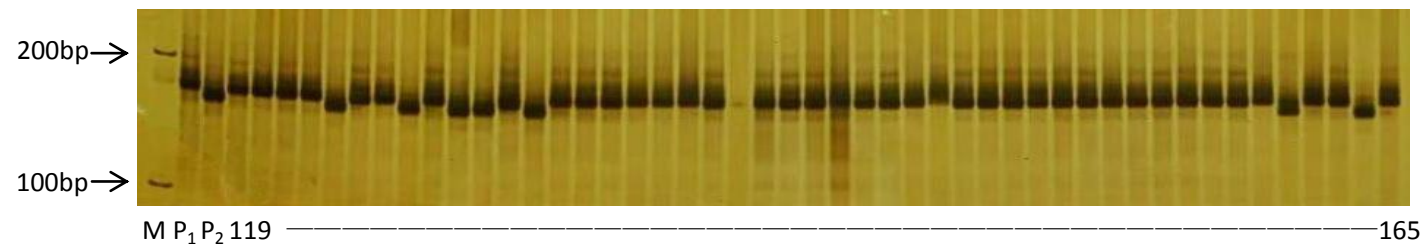

Supplement: Figure S4 — The allelic diversity of Cldw-1 gene in different watermelon germplasms using marker dCAPS3 (A) and Indel1 (B). M represented marker (100bp DNA ladder), P1 represented the normal height line WT4, P2 represented the dwarf line WM102, the number from 1 to 165 was corresponded to the natural watermelon germplasms in accordance with the code in Table S1 . [file Image_4.pdf]
