# Supplementary material for: A Single Nucleotide Deletion in an ABC Transporter Gene Leads to a Dwarf Phenotype in Watermelon
Source: Front Plant Sci. 2019 Nov 13;10:1399. doi: 10.3389/fpls.2019.01399 (PMC6863960; doi:10.3389/fpls.2019.01399)
Supplement: Figure S2 — The genomic sequence and exon-intron structure of ClDW-1 gene. The highlight sequences were indicated as exons. [file Image_2.pdf]

ATGGCGGAACCAGCAGCAGAGCCTAAGGCATTACCCGAACCCGAAAAGAAGAAGGAACAGAGCCTTCCT
TTTTACCAACTCTTCTCTTTTGCAGACAAATATGACTGGTTTCTCATGATCCTCGGTAGTTTCGGCGCC
ATTGTCCACGGCTCTTCCATGCCTGTTTTTTTCCTTCTTTTCGGCGAAATGGTTAACGGCTTCGGCAAA
AACCAATCCAATTTTCACAAAATGACAGCAGAAGTCTCTAAGGTTTTTCTCAATTTCAAACACAATTTC
TCTGTTTTTATCATTCTTCTGGTCTTTAGATCTCAGATCCGATTGTTCTTTTTTTTCTCTGTTTTGTTT
TCTTTAATTTGCAGTATGCTCTGTATTTTGTCTACTTGGGTCTCATTGTCTGTTTCTCATCCTACGCCG
GTAAGTAAACAAAATCCTCTGTTTTTTTTTTTTTTTCCCCTTCTCTCTTTATAACCCAAAAAAACAGAG
TTGATTAAATTTGCTGTTCTTCGTGTACAGAGATCGCATGTTGGATGTACACAGGGGAGAGGCAAGTGA
GTACATTGAGAAAGAAGTATTTAGAAGCTGTTTTGAAACAAGATGTTGGATTCTTCGACACTGACGCCA
GAACAGGGGATGTTGTTTTCAGTGTTTCAACAGACACTCTTCTTGTACAAGATGCAATCAGCGAGAAGG
TAACTTTTCTTAATTTCTTGTTTCCAAAAGGGAGAAAAATCTAATAGTAGCTAGTGTGGGGGCAAAACG
GATTACTTCATTCTGCAAAATGCCCATCTGGGTAGTTGCTATTTCATTAATTAAAGCCAATATGCGCAC
ATTAGCAAGATGGATTTTAACATGCACGTCTACAGAGCACCGCCATTAATACAGTGATTCTTGACCATG
AATAAGAATCTTTCTTCTTTTGTTGAATTGGGTTTTGATTTTGTTTAGGTGGGGAACTTCATTCACTAT
CTTTCGACGTTTCTAGCTGGATTGGTTGTGGGTTTTGTGTCAGCATGGAGACTAGCTCTTCTGAGTATA
GCAGTAATTCCAGGAATCGCTTTTGCTGGTGGTTTATATGCTTATACTCTCACTGGACTTACTTCAAAG
AGTCGAGAATCCTATGCCAATGCTGGCATAATTGCCGAACAGGTGAAACTTTTTTCCCCTTCTTTTCTA
TTTTTGGCTGCAATGTTAAGATTGAACTCTCTTTCACTGTTCTGTTTTGCCTTTGATCTTTAAAAGCAC
TTTCAAAATCTACCAGCCTGGCTTTGGTACTTAGCAACATGTTATCATGATGTTTATTCATGTGAAACA
AACTTTATCATCATTTTTGTTATTAATTTCATTCTAATCTTCATTTCCAAATGTTACTCAAGTTGATTC
TTTTTCACTTTTTGGGTCAAACCCACAGTAAAGTTCATTGAAGCAAGAATCACATGTCATGTTTTTTCA
TTGCAAAACTTCCAAGAACATAGCATGATATGTTTCATAAACGTGTTCCTATTAGGCGATTGCTCAAGT
TCGAACTGTTTACTCTTACGTTGGAGAAAGCAAAGCCCTGAATTCCTATTCAGATTCAATTCAAAACAC
ATTGAAAATTGGTTACAAGGCAGGAATGGCTAAGGGGTTGGGTCTAGGTTGTACCTATGGCATTGCTTG
TATGTCTTGGGCACTTGTCTTCTGGTATGCTGGAGTCTTCATCCGGAACGGCCAGTCCGATGGCGGCAA
AGCCTTCACTGCTATATTCTCCGCCATCGTCGGTGGCATGTAAGACTCAGGAAATTAGAAACTCTGATT
TAACCTACATCAACATCAAAGTTTCTGTTTTTTTGAATGTGGTTTTGTGTAATGCTATAGGAGCTTGGG
GCAGTCATTTTCCAATTTGGGAGCATTCAGCAAAGGGAAAGCAGCAGGGTACAAATTGATGGAGATTAT
CAAACAGAGGCCTACAATTATTCAAGACCCATTAGATGGGAAATGTTTGGGTGAAGTTAATGGCAACAT
TGAGTTCAAAGATGTCACCTTTAGCTATCCATCCAGGCCGGACGTGATGATCTTTAGGGATTTCTCCAT
TTTTTTCCCTGCCGGGAAGACGGTGGCTGTCGTTGGTGGTAGCGGTTCCGGGAAAAGCACTGTTGTCTC
TCTCATTGAAAGATTCTACGATCCCAATCAAGGTTATTAACAGCCAGTGTATATAACTAACACACTACA
TTGCTAGCTTGTGAATTGTTCTGATTACTATTTTCTCTAAAAAACAGGGCAGGTTTTACTAGACAATGT
AGACATCAAGACACTGCAATTGAAATGGCTAAGAGATCAAATTGGTCTAGTGAATCAGGAACCGGCTCT
GTTTGCAACCACCATATACGAAAACATTCTCTACGGAAAACCCGATGCGACGATGGCGGAGGTGGAAGC
TGCTGCGGCTGCAGCCAACGCTCATAGCTTCATCACATTGCTCCCCAATGGCTACGACACCCAAGTTAG
TACAATCCTTCAATGGGTTTCAATTTCAACACAATTTTGTGTTTTTTTTTCCATGACATGACAGTTCTT
AAATCAAAACCCAGGTGGGCGAAAGAGGATTACAACTCTCCGGTGGACAAAAACAGAGGATTGCCATAG
CCAGAGCAATGTTGAAGAACCCCAAAATCCTTCTCCTCGATGAAGCCACCAGCGCACTCGATGCAGGCT
CTGAGAGCATAGTTCAAGAAGCTTTAGACCGTTTAATGGTCGGAAGAACAACAGTGGTGGTTGCACATC
GATTATCAACAATAAGAAATGTCGATTCAATTGCAGTTATACAACAGGGGCAGGTAGTCGAAACAGGAA
CTCACGAAGAACTAATCGCTAAATCCGGTGCTTATTCCTCGTTAATTCGATTCCAAGAAATGGTTCGAA
ATAGAGAATTCTCAAACCCATCGACCCGTCGAACACGATCTTCACGACTGAGTCATTCGTTATCGACAA
AATCGTTAAGCCTCCGCTCCGGCAGCCTCAGGAATTTGAGTTATTCGTATAGCACCGGCGCCGATGGGA

GAATCGAAATGGTCTCAAACGCCGAAACTGACAGGAAAAACCCAGCCCCCGATGGCTATTTCATTCGTC
TTCTTAAACTGAACGGCCCCGAATGGCCGTATTCGATAATGGGCGCTGTTGGGTCTGTTCTCTCTGGTT
TCATCAGTCCAACATTTGCCATTGTTATGAGTAACATGATCGAGGTCTTCTATTACAGAAATTCATCCG
CCATGGAAAGGAAAATCAAGGAATTCGTATTCATCTACATTGGAATTGGGGTTTACGCAGTGGTAGCTT
ATCTAATTCAGCATTATTTCTTTACAATCATGGGTGAGAATCTCACTACCAGGGTCAGAAGAATGATGC
TCGCAGGTAAAATATCAATCAATAAGCTTCATACTTCCCAAGTTTTTTTTTTTTTTTTTTTTTTTTTAA
TTTATAAATCCTTAATATCAAAATAATGTGAAATCCTCTGTTTTTTTTTTTTTTTTTTTTTTTTCTCTA
TAGCGATTTTGAGGAATGAAGTGGGATGGTTCGATGAAGAAGAACACAACTCAAGTTTAGTAGCAGCAA
GATTGGCTACTGATGCAGCTGATGTAAAATCCGCCATTGCTGAAAGAATATCAGTGATACTACAAAATA
TGACTTCACTCTTCACTTCTTTTATAGTTGCATTTATTGTTGAATGGAGAGTTTCTCTTCTCATCCTTG
CAGCCTTCCCTCTACTGGTTCTTGCCAACATGGCTCAGGTAACCAAAACCCACTTCCCAAAACTTCATT
AATTTCTATTCCAAAGCATTTTAAAACAACCCACATAAAAAAGATTAGTTTTTTTTTTTTAAGGAAGTT
TTTCATTGGAAAGCTAGGGGTTTCCTTGCATGAGTTGACCCTAGTCTAAAAAGGGTGGCATAAGGGGCT
GCAGTTAGTGAAAATTTAATTTTGAAGAAATGGGTCAGATTCTCTGAACTTTGCTTTTTACAGCTCTTG
GAACATAACTTCACTGCATACTAAGTCCTATTCTTCTATTACGCGACAAAGAACCTGAAATATTTAACT
CTTACCATCTCTGTGCTCTGCACGTGGTTCATCCCACTGCCTGCTCTGCACTAACTTCTGAAAACTTTG
CAGTGATAAACATTAAAGAAATAACTCTGAAAATTCATACCTTTTAATATAATAAAAATACATGACAAA
GACAAACAAGCGAGGAAACTCTTCGAAATTCCAACAAGATTCAAACTTTACTAAAAAATTTCTTACCCC
CCTTTCTTTTTTCATGTGGAAAGTCCCTGGGTTTTGCTTTGTTTTTGTTTTAGCTTTTACTTCGAGCCC
AATGCCTATATTTTTCTTGGTTTCTCCGTTTTTATTAGTTTTTTTATTCTATACATACACCGACTCTTC
TGCATCTCTGCAGTATTTTTATCAGCTCTCTGTTTTTCTTTTTTTTCTTCTGAAAGCTTCAAGTTTTGA
CCATCTTTCTCTCCCTTGAAATCCGTGCTCTCTCTGGTTTCTCTGGCTTTTGCCAGATACCCCATGTGA
AAAACTGCAGCTAATTTCTCACTCCCCGTGATCTTCATTCATATAATATAAAGTTTTCAAACTTAGCTC
TTCACATTTTTCCATAAAAGAGAAGGGAAAAAAAGGGGTATAGTATAGTATTTTTGCTGCAAAGTTCAG
TAACTGACGGACTTCATTATACATTGTCGTCTTTTGGCAATTTCTCTGAAAGTGATTAAATCTAATGGG
AGGGGGTTCCAAAAAATGGGGTTCGTTTTCAAACTATAGTGTACTGATCTCTGACCATTTGTCTAGTGT
TGAACAGTAGTGTAAAATTTGCGCATGCTTCTAATACAGTGAAAGTTACGACTGATTGCAAAAGGACAT
TACTTTCTGCTACCTATAAATGTCTCTCTCATTGTTCCTTTTTTCATGTTTAGGTAAAATGATCCTCTT
TTAATTACCCCCCCCCCCCCCCCCCCCCCCCCACTAACCCCCCCCCCCCCCCCCCCCCCCCCCCAATTG
TTTGAAACTCATACATCAAACAAAGCTTTGTTCTTAATTGAACTTTGATCACTGAGCTTGATTTGAATT
AGGCTAATCTAATTAGAGATGAGTTTAACGACAGAAGTTGAACATTGTTATTTATTATGCAGCAACTTT
CTTTAAAAGGATTTGCTGGGGATACTGCTAAGGCCCACGCAAAGACAAGTATGATTGCAGGGGAAGGTG
TGAGTAATATCAGAACAGTGGCTGCCTTCAATGCTCAAGACAAGATCCTTTCTCTGTTCCGCCACGAGC
TTCGTGTCCCACAACGACAGAGTCTCCGCCGGAGCCAAACGGCAGGTGTCCTTTTCGGTATCTCACAGC
TAGCTTTATATGCCTCTGAAGCTTTAGTTCTATGGTATGGTGTCCATTTGGTCAGCAATGGTGGATCAA
CATTCTCCAAAGTGATCAAAGTCTTTGTTGTCTTGGTTGTTACTGCCAATTCTGTGGCGGAGACTGTTA
GTCTTGCTCCTGAGATCATTAGAGGTGGTGAATCAATTGGTTCTGTTTTCTCAATTCTCGATCGCCCGA
CGAGGATCGACCCTGATGATCCTGAGGCAGAGACTGTTGAGACGCTTCGTGGGGAAATTGAACTCCGAC
ATGTTGACTTCGCATACCCGTCTCGACCTGATGTCATGGTGTTTAAGGATCTCAATTTGAGGATCAGGG
CTGGTCAGAGCCAGGCATTGGTTGGAGCCAGTGGATCAGGGAAGAGTTCAGTGATTGCTTTGATTGAGA
GATTCTACGATCCGCTTGCCGGGAAAGTTATGATCGACGGGAAGGACATTCGACGCCTGAACTTGCAAT
CCTTGAGGCTGAAAATTGGTTTGGTACAGCAAGAGCCAGCCTTATTTGCAGCCAGCATTTTTGACAATA
TTGCATATGGAAAAGATGGAGCAACAGAGGCAGAAGTAATAGAGGCAGCAAGAGCAGCCAATGTGCATG
GCTTTGTAAGTGGACTTCCTGATGGCTACAAGACACCAGTTGGGGAAAGAGGCGTTCAACTCTCTGGTG

GCCAAAAACAACGCATAGCTATTGCCAGAGCTGTTCTCAAGGACCCCACAATCCTCCTCCTCGACGAAG
CTACCAGTGCTCTCGACGCCGAGTCCGAGTGTGTGCTGCAAGAAGCTCTCGAAAGGCTCATGAGGGGCC
GGACCACGGTGGTCGTGGCACATCGCCTGTCCACAATCAGGGGTGTGGACTGCATTGGCGTGGTCCAAG
ACGGTCGCATTGTCGAGCAAGGCAGCCATAATGAGCTGCTAAGCCGAGCTGAGGGGGCTTACTCAAGGC
TCTTGCAGCTGCAACACCAACACATATGA
